# Adsorption of Heavy Metals by Graphene Oxide/Cellulose Hydrogel Prepared from NaOH/Urea Aqueous Solution

**DOI:** 10.3390/ma9070582

**Published:** 2016-07-16

**Authors:** Xiong Chen, Sukun Zhou, Liming Zhang, Tingting You, Feng Xu

**Affiliations:** Beijing Key Laboratory of Lignocellulosic Chemistry, Beijing Forestry University, Beijing 100083, China; cx3130370@bjfu.edu.cn (X.C.); zhousukun@bjfu.edu.cn (S.Z.); zhanglimin@bjfu.edu.cn (L.Z.); youtingting0928@bjfu.edu.cn (T.Y.)

**Keywords:** heavy metal ions, hydrogel, cellulose, graphene oxide, NaOH/urea

## Abstract

By taking advantage of cellulose, graphene oxide (GO), and the process for crosslinking using epichlorohydrin (ECH), we propose a simple and novel method to prepare GO/cellulose hydrogel with good potential to adsorb metal ions. GO nanosheets containing carboxyl and hydroxyl groups were introduced into the surface of the cellulose hydrogel with retention of the gel structure and its nanoporous property. Due to the introduction of GO, the GO/cellulose composite hydrogels exhibited good compressive strength. Adsorption capacity of Cu^2+^ significantly increases with an increase in the GO/cellulose ratio and GO/cellulose hydrogel showed high adsorption rates. The calculated adsorption capacities at equilibrium ($q_{e}^{\mathrm{cal}}$) for GO/cellulose hydrogel (GO:cellulose = 20:100 in weight) was up to 94.34 mg·g^−1^, which was much higher than that of the pristine cellulose hydrogels. Furthermore, GO/cellulose hydrogel exhibited high efficient regeneration and metal ion recovery, and high adsorption capacity for Zn^2+^, Fe^3+^, and Pb^2+^.

## 1. Introduction

Due to the industrialization process, the serious threat of heavy metal ions to the environment is a particular concern worldwide. Heavy metals are among the most common pollutants found in wastewater and can be accumulated in the environment and living tissues, causing various diseases and disordering of living organisms even at a trace level [1]. Thus, it is necessary and urgent to remove hazardous heavy metals from aqueous solutions. A variety of techniques have been developed, such as chemical coagulation, ion exchange, chemical oxidation/reduction, membrane separation, electrochemical techniques, and ultrafiltration [2]. However, these techniques have disadvantages, such as low efficiency, high cost, and generation of other waste products. Therefore, searching for more effective adsorbents is of immense interest in wastewater treatment [3]. 

Bioadsorption is considered to be a potential alternative to conventional technologies for the adsorption of metal ions from aqueous solutions [1]. A great deal of attention has been diverted toward the production of bioadsorbents from renewable resources, such as cellulose, starch, lignin, and agricultural wastes. These bioabsorbents have many advantages over conventional adsorbents, such as low cost, are biodegradable, eco-friendly, and highly efficient [4]. Especially, the hydrogels obtained from cellulose have spurred great interest in the adsorption of heavy metal ions from aqueous solutions, because of their particular physicochemical properties, such as the facility of the incorporation of different chelating groups into the polymeric networks, the internal porous structure, are eco-friendly, cost-effective, and have a high specific surface area [5]. Hence, as a typical soft matter, cellulose-based hydrogels have wide application in dye removal, adhesion, and ion adsorption [6,7]. The new mixture solvent of NaOH/urea aqueous systems makes it easy to prepare cellulose-based hydrogel [6]. This solvent has been suggested as an environmentally friendly system [8]. Nevertheless, most of the hydrogels suffer from a lack of adsorption capacity and mechanical performance. The poor adsorption and mechanical property of hydrogel has limited its further industrial applications [9].

Recently, some multifunctional hybrid materials based on GO, such as GO/ethylenediamine-triacetic acid (EDTA), GO/RNA, and GO/Fe_3_O_4_, have been successfully used as the absorbents [10,11,12]. GO has an extended layered structure with various functional groups (hydroxyl, carboxyl, and epoxy groups) in GO, which results in its interesting dispersibility and affinity to many pollutants in water [13]. Additionally, its huge surface area endows it with strong adsorption abilities much like carbon nanotubes [14]. Meanwhile, composite hydrogels are considered to be a simple way to improve the mechanical properties of hydrogels with the addition of reinforcing organic/inorganic fillers, for example, clay, GO, carbon nanotubes, etc. [9]. GO have been studied as the reinforcing fillers because of their high aspect ratio, excellent modulus, and intrinsic strength. Moreover, containing numerous oxygen functional groups on their surfaces, GO could have strong interaction with polar polymers [15]. Therefore, GO could enhance not only the strength, but also the adsorption ability of porous GO/cellulose hydrogels. In fact, there is already some previous research devoted to the composite of cellulose and GO. By the process of freeze-drying, Zhang et al. prepared cellulose/GOS aerogel with high mechanical strength and good thermal stability [15]. Zhang et al. reported that cellulose/GO composites were prepared by mixing dissolved cellulose with GO, followed by reducing with hydrazine hydrate, which exhibited good triazine pesticide adsorption properties [16]. However, to the best of our knowledge, there has been no attempt, and the systemic investigations have been reported, on hydrogel prepared from GO and cellulose in the NaOH/urea aqueous system by using epichlorohydrin (ECH) as cross-linker.

In view of these facts mentioned above, we show a simple, novel, and environmental-friendly preparation of cellulose-based hydrogel by incorporating GO into the cellulose matrix using NaOH/urea aqueous solution as the processing solvent. Due to the introduction of GO, the GO/cellulose composite hydrogels exhibited good adsorption capacities for heavy metal ions and high compressive strength. This study provided a highly efficient bioadsorbent for the removal of heavy metals from aqueous solution.

## 2. Results and Discussion

### 2.1. Characterization of GO/Cellulose Hydrogel

**Figure 1:**
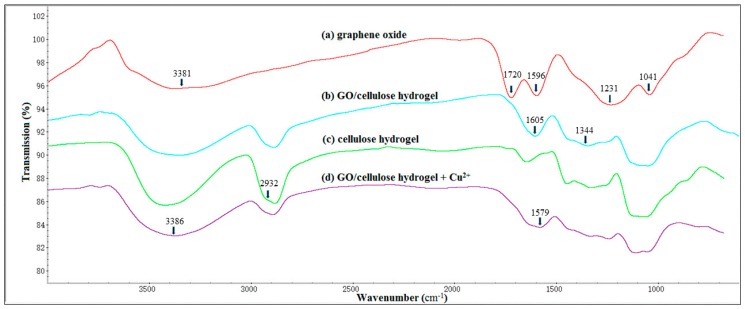
FTIR spectra of GO, GO/cellulose hydrogel, cellulose hydrogel, and Cu(II)-loaded GO/cellulose hydrogel.

FTIR was used to expound the characteristics of GO, GO/cellulose hydrogel, cellulose hydrogel, and Cu(II)-loaded GO/cellulose hydrogel, as shown in Figure 1. GO exhibited a strong absorption band at 3381 cm^−1^, which corresponds to a characteristic band of –OH. The absorption band at about 1596 cm^−1^ is assigned to the aromatic C=C, while the absorption bands at 1720, 1231, and 1041 cm^−1^ are assigned to the stretching vibrations of carboxy (C=O), epoxy (C–O–C), and alkoxy (C–O) groups, respectively [17]. In the spectrum of GO/cellulose, the absorption band attributed to epoxide groups disappeared, while the absorption intensity for alkoxy groups at 1041 cm^−1^ increased, suggesting the successful conversion of epoxide groups into alkoxy groups. The intensity of absorption peaks at 3381 cm^−1^ was diminished due to the decrease of hydroxyl groups when compared to that of GO. The results indicated that the crosslinking reaction of cellulose and GO in NaOH/urea aqueous solution with ECH occurred. The symmetric stretching vibration of CH_2_ is visible at 2923 cm^−1^ and 2873 cm^−1^ (spectra b, c and d), in agreement with the literature data [18]. Compared with the cellulose hydrogel without GO (spectrum c), two distinct band observed at 1605 cm^−1^ and 1344 cm^−1^ in the GO/cellulose hydrogel (spectrum b) can be attributed to COO^−^ stretching and bending, respectively [19]. Here, we present evidence for the existence of the carboxyl groups of GO in the hydrogels. After copper ion adsorption on the GO/cellulose hydrogel (GO/cellulose hydrogel + Cu^2+^, spectrum d), the absorption bands of COO^−^ groups at around 1605 cm^−1^ shift to 1579 cm^−1^. This can be attributed to the formation of the coordinated COO^−^ and Cu^2+^ complexes [1]. The O–H band absorption peak was observed to shift to 3386 cm^−1^ when the GO/cellulose hydrogel is loaded with Cu^2+^. It seems that this functional group participates in metal binding [20].

**Figure 2:**
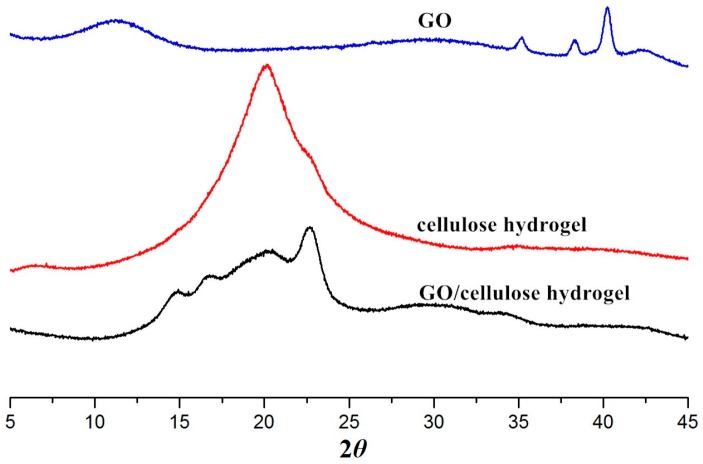
XRD patterns of GO, cellulose hydrogel, and GO(20)/cellulose(100) hydrogel.

To obtain information about the crystalline structure of the GO, cellulose hydrogel and the GO/cellulose hydrogel, the x-ray diffraction (XRD) patterns of these samples were measured and are shown in Figure 2. GO exhibits a characteristic diffraction peak at 2θ = 11.4°, resulting from its (002) crystal planes [21]. The XRD pattern of GO contains a peak at around 41°, which is related to the (100) plane of the graphite [22]. Cellulose hydrogel displays the diffraction peaks at 2θ = 20.1° and 22.5°, which correspond to the (110) and (200) planes of cellulose II crystalline form, respectively. GO/cellulose hydrogel exhibits three distinct peaks at 2θ = 14.1°, 20.1°, and 22.5°, which are assigned to the ($1\bar{1}0$), (110), and (200) planes of crystalline form of cellulose II, respectively [23]. However, the peaks of GO/cellulose hydrogel moved from 12.1° to 14.1°. The cross-linking reaction of ECH with GO and cellulose may be due to shrinkage of the ($1\bar{1}0$) planes causing this peak to move to higher angles [24]. The results indicate that the structure of cellulose I was destroyed in aqueous NaOH/urea and transformed into cellulose II. In contrast, the GO/cellulose hydrogel generates only the characteristic peaks of cellulose with no characteristic peak of GO. These findings can be explained as the high dispersibility of the GO sheets in the GO/cellulose hydrogel due to the bond interactions between the cellulose molecules and the GO sheets, so that the periodic interlayer spacing between the GO sheets disappeared [25].

**Figure 3:**
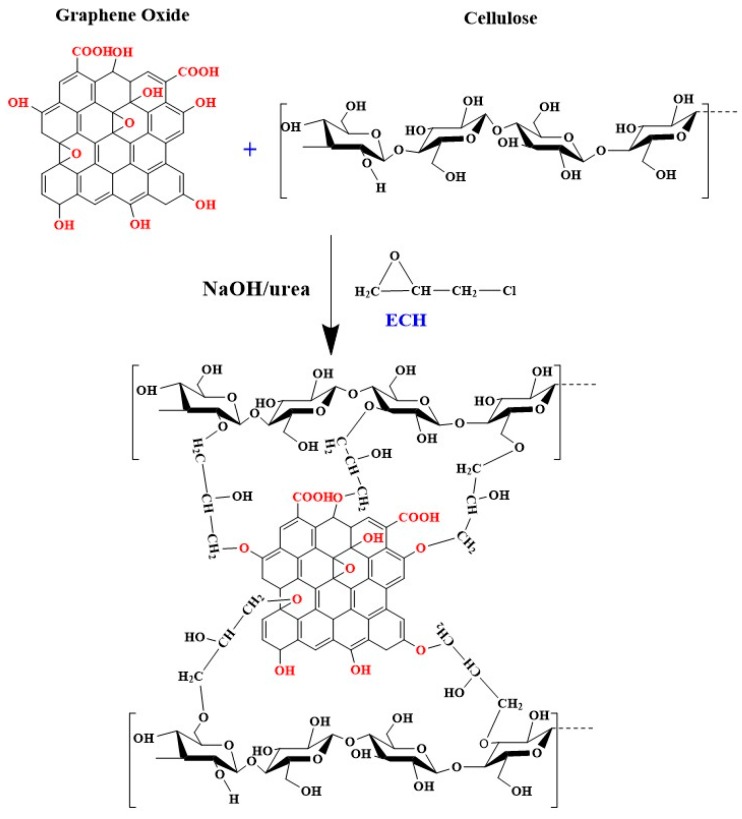
Proposed mechanism for cross-linking reaction of ECH with GO and cellulose.

To sum up, the mechanism for cross-linking reaction of ECH with GO and cellulose in NaOH/urea solution is schematically illustrated in Figure 3. The hydroxyl groups of the cellulose were cross-linked covalently with epoxy and hydroxyl groups of the GO through nucleophilic attack of the alcoholate anion to form a monoethers of chloropropanediols and a new epoxide formed by chloride displacement, leading to the completion of the cross-linking [19].

**Figure 4:**
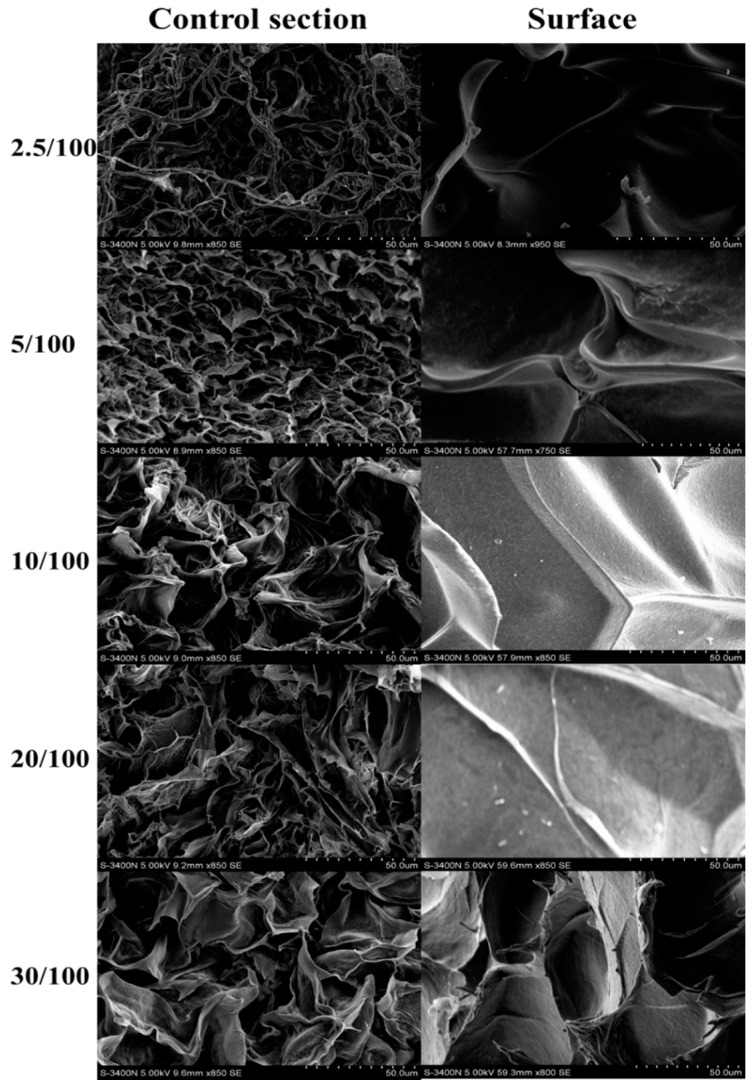
SEM images of GO(*x*)/cellulose(100) dry hydrogels with *x* = 2.5, 5, 10, 20, and 30, respectively. Cross-section images were taken inside the gel. Surface images were taken on the surface of the hydrogels.

**Figure 5:**
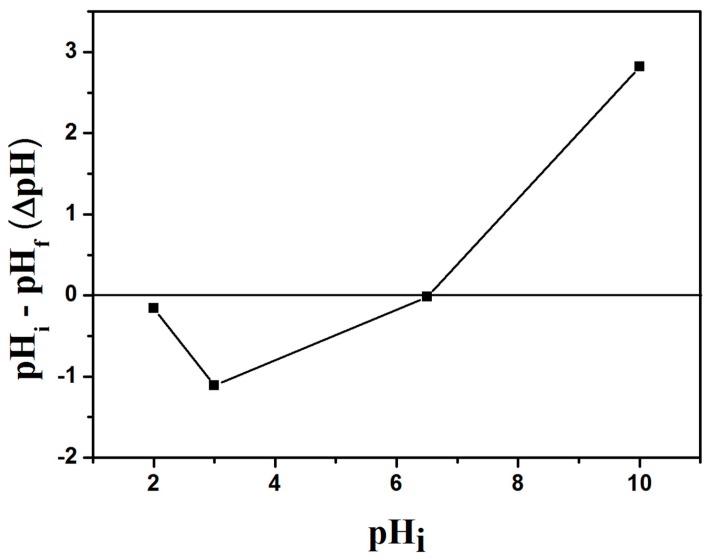
Point of zero charge of GO(20)/cellulose(100) hydrogel.

As can be seen in Table 1, the samples with different content of GO showed similar water content. The compressive modulus of the hydrogels increased to a maximum and then decreased, with the GO/cellulose ratios increasing from 2.5/100 to 30/100. At lower concentrations, this dependence of the compressive modulus on the content of GO is perhaps due to the chemical bond between the cellulose fibers and the surface of GO. However, GO also reduces macromolecular interactions which decrease the compressive strength [23]. So the GO(5)/cellulose(100) hydrogel possessed the higher compressive modulus. The value is much higher than those of cellulose-alginate hydrogel (30.9 kPa) [26], pure cellulose hydrogel (48 kPa) and cellulose/poly(*N*-isopropylacrylamide) hydrogel (58 kPa) [27]. The GO/cellulose hydrogel, in general, has a large specific surface area. Furthermore, it can be seen that, with increasing GO content, the Brunauer-Emmett-Teller (BET) surface areas and pore volume of the samples resulted in an obvious enhancement. This indicated that the electrostatic repulsions caused by the ionic character of the carboxylate anions (COO^−^) in GO had enlarged the space in the networks of hydrogels [19]. Compared with the GO(10)/cellulose(100) sample and the GO(30)/cellulose(100) sample, a notable reduction of the specific surface area, pore volume, and average pore size of GO(20)/cellulose(100) hydrogel was observed. One possible explanation was the formation of GO sheets on the surface and inner of GO(20)/cellulose(100) hydrogel, leading to the block of some pore structures [28]. Another possible explanation was the agglomeration of the graphene oxide sheets [29]. The SEM images of the GO(*x*)/cellulose(100) dry hydrogels are shown in Figure 4. The vast majority of the cross-sectional images of the inside of the gel showed a macropore architecture, indicating good miscibility between GO and cellulose. The cross-sectional images of the GO(2.5)/cellulose(100) sample exhibited a homogenous fine fibrillary structure because of the incomplete dissolution of cellulose. The surface of GO/cellulose hydrogels showed smooth morphology, which also indicated that the cellulose was miscible with GO. As shown in Figure 5, there was a distinct common point of intersection at the ΔpH = 0 line at pH*_i_* = 6.5, which was the pH_pzc_ of the GO/cellulose hydrogel. Hence, the hydrogel is positively charged at a pH below pH_pzc_ and negatively charged at a pH above pH_pzc_. The above results suggest that the electrostatic attraction between metal ions and the hydrogel surface should increase with increasing solution pH [30]. 

### 2.2. Adsorption Measurements

#### 2.2.1. Effect of GO/Celluloses Ratios, Cu(II) Solution pH, and Dosage on Cu^2+^ Uptake

**Figure 6:**
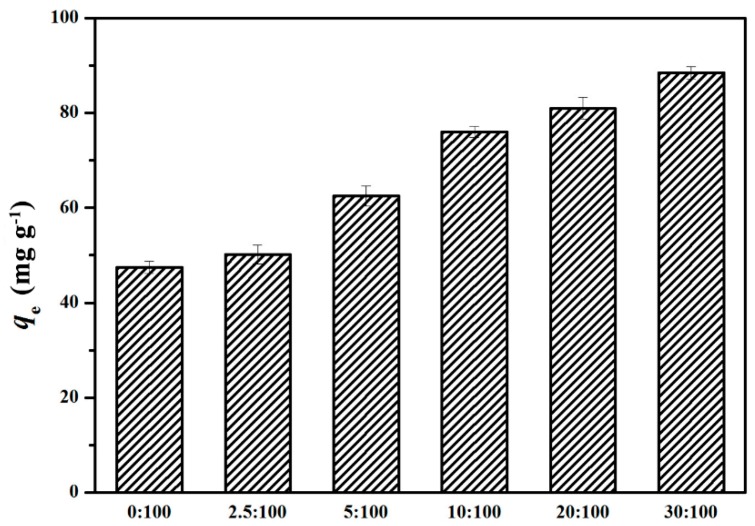
Effects of GO to cellulose ratios on Cu^2+^ ion adsorption on the hydrogel. The error bars represent standard deviations based on three measurements.

**Figure 7:**
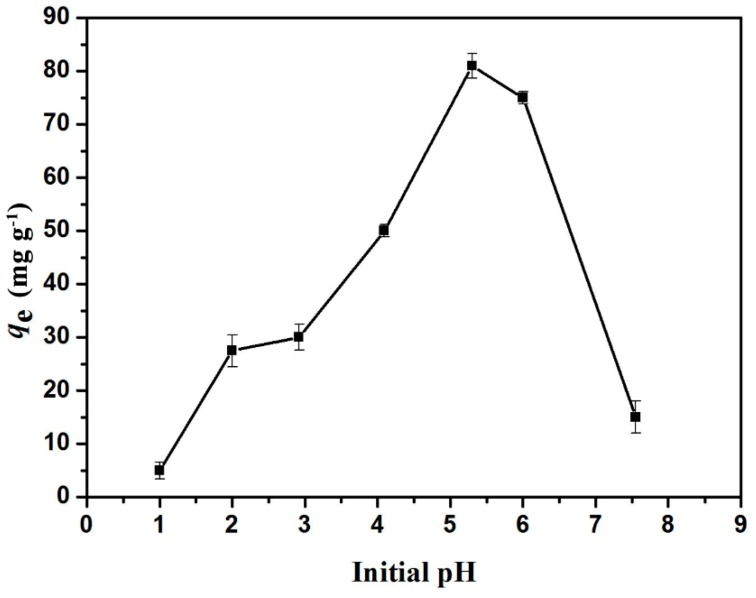
Effect of pH on the adsorption capacity. The error bars represent standard deviations based on three measurements.

**Figure 8:**
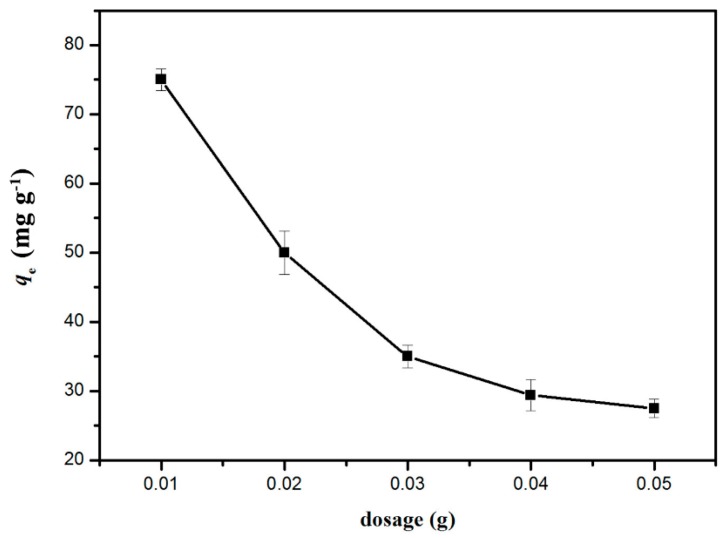
Effects of adsorbent dose on Cu^2+^ adsorption by the GO(20)/cellulose(100) hydrogel. The error bars represent standard deviations based on three measurements.

The effect of GO/celluloses ratio on Cu^2+^ uptake is shown in Figure 6. The adsorption capacity of Cu^2+^ was 47.5 mg/g at a GO/cellulose ratio of 0:100, and then increased to 88.5 mg/g as the ratio increased to 30:100. The adsorbent has a heterogeneous distribution of GO on the surface. The amount of sorbate which is adsorbed per unit weight of adsorbent at a given solution concentration is not proportional to the surface area, indicating that the characteristics of the surfaces of the GO/cellulose hydrogels are different in each case. This phenomenon should be attributed to more oxygenous functional groups being incorporated into the hydrogel as the GO/celluloses ratio increases, which increase the surface complexation, electrostatic attraction, and ion-exchange capability of bioabsorbent [31]. One problem with GO(30)/cellulose(100) hydrogel is incomplete cross-linking, probably because of the high ratio of GO/ECH. Thus, GO/cellulose hydrogel with a ratio of 20:100 was chosen in the following experiments. As shown in Figure 7, the experiments were carried out in the pH range 1.0–7.5. The adsorption capacities of Cu^2+^ increased as pH increased from 1.0 to 5.3. This is because the pH value affects the surface charge of the adsorbent. When the pH value increased, the negative charge of the adsorbent increased [32]. Above pH 5.3, the solution became turbid. Meanwhile, the GO/cellulose hydrogel displayed a sharp decrease in the uptake values when pH increased. The cause for the phenomenon could be the reduced solubility and precipitation of Cu^2+^ under alkaline condition [33]. Therefore, the optimum pH value for Cu^2+^ absorption onto GO/cellulose hydrogel was about 5.3. The effect of hydrogel dosage on the adsorption properties was investigated in the range 0.01–0.05 g, and the results are presented graphically in Figure 8. It was found that *q*_e_ decreases from 81 to 27.5 mg·g^−1^ with an increase in adsorbent mass from 0.01 to 0.05 g. The reason for this phenomenon is attributed to the unsaturation of adsorption sites through the adsorption process. Another reason may be the particle interactions, such as aggregation, resulting from high adsorbent concentration. Such aggregation would lead to a decrease in the total surface area of the adsorbent [34]. 

#### 2.2.2. Adsorption Kinetics Studies

**Figure 9:**
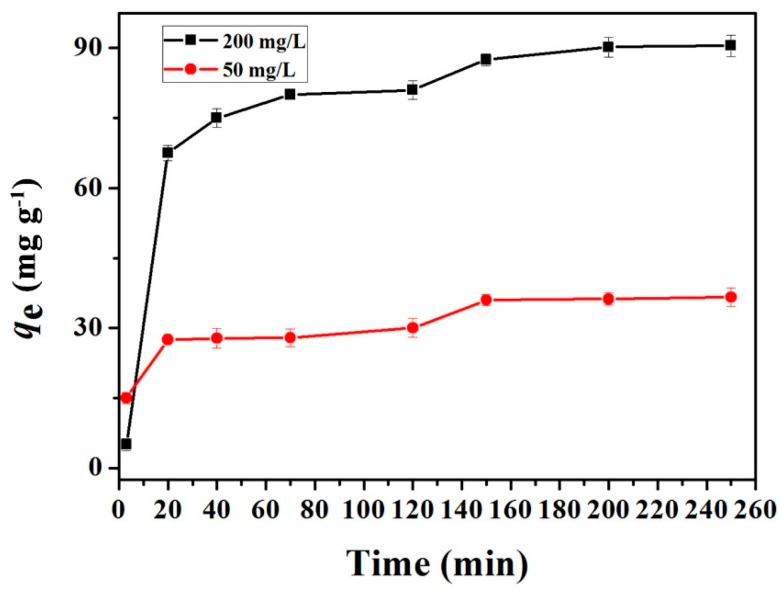
Adsorption of Cu^2+^ on the GO(20)/cellulose(100) hydrogel as a function of contact time. The error bars represent standard deviations based on three measurements.

The copper(II) adsorption capacities of the GO(20)/cellulose(100) hydrogel were measured as a function of contact time, and the results are shown on Figure 9. The adsorption capacities of Cu^2+^ increased rapidly at short time scale and the adsorption process attains equilibrium within 150 min, indicating that plenty of readily-accessible sites were available for a rapid adsorption [35]. Adsorption kinetic provided important information about the mechanism of Cu^2+^ adsorption onto GO/cellulose hydrogel, which was necessary to describe the adsorbate-adsorbent interactions. The Lagergren’s pseudo-first-order and pseudo-second-order models are the most commonly used models. The linear pseudo-first-order kinetic model (Equation (1)) and pseudo-second-order model (Equation (2)) are expressed by the following equations:

ln(*q*_e_ − *q*_t_) = −*k*_1_*t* + ln*q*_e_ (1)

*t*/*q* = *t*/*q*_e_ + 1/*k*_2_*q*_e_^2^ (2)

where *q*_t_ and *q*_e_ are the amounts adsorbed (mg·g^−1^) at time *t* (min) and at adsorption equilibrium, respectively, *k*_1_ (min^−1^) is the kinetics rate constants for the pseudo-first-order model, and *k*_2_ (g·mg^−1^·min^−1^) is the kinetics rate constants for the pseudo second-order model. The values of ln(*q*_e_ − *q*_t_) obtained from the kinetics experimental data. The kinetic models are examined by linear plots of ln(*q*_e_ − *q*_t_) against *t* and (*t*/*q*) against *t*, respectively. The boundary conditions are *q* = 0 at *t* = 0, and *q* = *q* at *t* = *t*. Table 2 lists the characteristic parameters and regression coefficients obtained from the first- and second-order kinetic models. 

By comparing the two kinetics models, the higher correlation coefficients (*R*^2^ in Table 2) were obtained for the pseudo-second order kinetic model, and the calculated data ($q_{e}^{\mathrm{cal}}$ in Table 2) from the pseudo-second-order kinetic model generally deviate less from the experimental data. These results indicate that the adsorption system is well-represented by the pseudo-second-order kinetic model, and the rate of occupation of adsorption sites is proportional to the square of the number of unoccupied binding sites [36]. Therefore, the adsorption of Cu^2+^ by bioadsorbent is dominated by a chemical adsorption process. The interaction may occur between the COO^−^ and the Cu^2+^ ions, which means that the adsorption mechanism of GO/cellulose hydrogel is ion exchange [4]. The calculated adsorption capacities at equilibrium ($q_{e}^{\mathrm{cal}}$) for GO(20)/cellulose(100) hydrogel was 94.34 mg·g^−1^, which was much higher than that of pristine cellulose hydrogels [37]. The value is much higher than those of acrylic acid-grafted and acrylic acid/sodium humate-grafted bamboo cellulose nanofibers (46.53 and 45.38 mg/g, respectively) [38] and cellulose/chitosan composite microspheres (65.8 mg/g) [39].

#### 2.2.3. Adsorption Isotherm Studies

**Figure 10:**
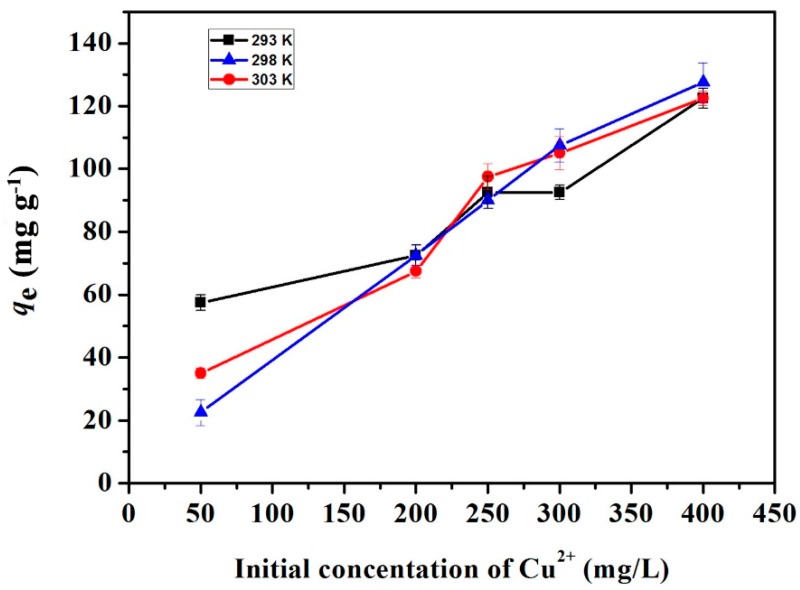
Adsorption of Cu^2+^ on the GO(20)/cellulose(100) hydrogel as a function of Cu^2+^ concentration. The error bars represent standard deviations based on three measurements.

The adsoption isotherms of the GO(20)/cellulose(100) hydrogel for Cu^2+^ ion are presented in Figure 10. Cu^2+^ ion uptakes of the GO/hydrogel cellulose increased linearly with increasing Cu^2+^ concentration, suggesting that the adsorption capacity was dependent on the amount of metal ions. To further understand the process, the adsorption data were subjected to Langmuir (Equation (3)) and Freundlich (Equation (4)) models for simulation. The Langmuir model is a widely-applied model based on the assumption of monolayer adsorption onto a surface containing a finite number of adsorption sites of uniform strategies of adsorption without transmigration of adsorbate in the plane of the surface [40]. The Freundlich model is derived by assuming an exponential decay energy distribution function inserted in the Langmuir equation with the amount adsorbed being the summation of adsorption on all sites with different bond energies [41].

*C*_e_/*q*_e_ = 1/*Q*_max_*b* + *C*_e_/*Q*_max_ (3)

ln*q*_e_ = ln*k* + 1/*n* × ln*C*_e_ (4)

where *q*_e_ (mg/g) is the amount of Cu^2+^ ion adsorbed at equilibrium, *C*_e_ (mg/L) is the concentration of Cu^2+^ ion, *Q*_max_ (mg·g^−1^) and *b* (dm^3^·mg^−1^) are the Langmuir equation parameters; *k* is the Freundlich isotherm constant (L·mg^−1^), and *n* is the Freundlich factor.

The parameters of the simulation are all listed in Table 3. The correlation coefficients (*R*^2^) of the linearized Langmuir equation are lower than that of the Freundlich equation. The GO/cellulose hydrogel was described better with the Freundlich model than with the Langmuir model, which reveals that the bioadsorbency to Cu^2+^ ions is mainly through parallel π–π stacking interactions and form multilayer adsorption [13]. The presence of such heterogeneous adsorption sites may be the reason for the better applicability of the Freundlich isotherm [37]. Isotherms with *n* > 1 are classified as L-type isotherms reflecting a high affinity between adsorbate and adsorbent and is indicative of chemisorption [38].

#### 2.2.4. Repeated Use of Hydrogel and Adsorption of Other Hazardous Metals

**Figure 11:**
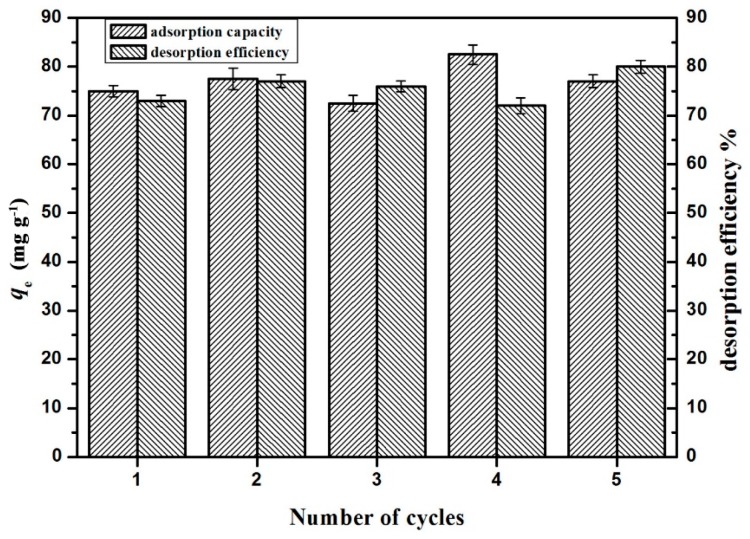
Effect of recycling bioadsorbents on Cu^2+^ adsorption (initial concentration 200 mg·L^−1^; initial pH of solution 5.3, temperature, 298 K; contact time, 120 min). The error bars represent standard deviations based on three measurements.

**Figure 12:**
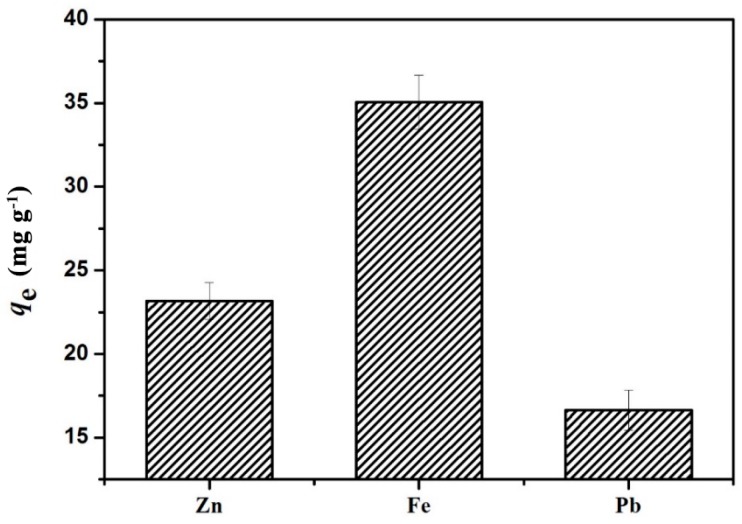
Adsorption amount of various metals on GO(20)/cellulose(100) hydrogels. The error bars represent standard deviations based on three measurements.

The effect of five consecutive adsorption-desorption cycles on the efficiency of the adsorption of Cu^2+^ on GO(20)/cellulose(100) hydrogel was studied, and the results are presented in Figure 11. As shown in Figure 11, no noticeable losses were observed in the adsorption capacity or desorption efficiency of GO/cellulose hydrogel as the number of cycles increased. In 1 M HCL solution, the protons compete with metal ions for carboxyl groups, which are responsible for the easy desorption of metal ions. This, again, confirms that the main adsorption mechanism is ion exchange [1]. During the regeneration process with NaOH solution, COOH groups were converted to COO^−^ groups which exhibited stronger affinity to Cu^2+^ [38]. The present study further revealed the advantage of GO/cellulose hydrogel which allowed for excellent reusability. The adsorption measurement was also performed on Zn^2+^, Fe^3+^ and Pb^2+^ ions (Figure 12). The *q*_e_ value was different for each ion and was in the order of Fe^3+^ > Zn^2+^ > Pb^2+^. The GO/cellulose hydrogel sufficiently adsorbed all of the metals tested, suggesting that the GO/cellulose hydrogel is a general-purpose bioadsorbent.

## 3. Materials and Methods

### 3.1. Materials

Cellulose with DP of 385 (cotton linter pulp) was supplied by Hubei Chemical Fiber Co. Ltd. (Xiangfan, China). The α-cellulose content in cotton linter pulp was more than 95%. Length and width of cellulose fiber were measured from 362 to 619 μm and 18 to 36 μm, respectively. All cellulose samples were shredded into pieces and distributed, and vacuum dried at 60 °C for 24 h to remove adsorbed water before use. All chemicals of analytical grade were obtained from Beijing Chemical Co. Ltd. (Beijing, China) and used without further purification. The standard solutions (1000 μg/mL) of Zn (II), Fe (III), and Pb (II) were purchased from the National Institute of Metrology (Beijing, China). The graphite was supplied by Jinrilai Graphite Co., Ltd. (Qingdao, China).

### 3.2. Preparation of GO

GO was prepared from natural graphite by a modified Hummers method [42]. Briefly, graphite (5.0 g), sodium nitrate (2.5 g), and concentrated sulfuric acid (95%, 115 mL) were consistently mixed in an ice bath for 1 h. While maintaining vigorous agitation, 15 g KMnO_4_ was slowly added to the suspension. The rate of addition was carefully controlled to keep the temperature of the reaction mixture below 5 °C. Next, the mixture was placed in a 45 °C water bath and kept at that temperature for 30 min, followed by the slow addition of distilled water (230 mL) to keep the solution from effervescing. The resulting solution was placed at well below 70 °C–80 °C for 30 min. With progression of the reaction, the color turned into light brownish. After further treatment with H_2_O_2_ (30%, 25 mL), the filtered cake was washed with 5.6 L of 10% HCl and then with considerable water. After drying under vacuum for 24 h, the grey-black powder of GO was obtained.

### 3.3. Preparation of GO/Cellulose Hydrogel

A solution of 4.0 wt % cellulose in NaOH/urea aqueous solution was prepared according to the previous work [43]. GO was dispersed into the 7.0 wt % NaOH/12.0 wt % urea aqueous solution precooled to −12.6 °C for further ultra-sonication for 1 h. Cellulose (2 g) was added in the suspension (50 mL) and stirred for 15 min at 5000 rpm. Then, 6 mL ECH, as a crosslinking agent, was added dropwise to the GO/cellulose mixture. After completion of ECH feeding, the resultant mixtures were stirred at 25 °C for 30 min to obtain a homogeneous solution, and then kept at 25 °C for 48 h in a water bath to transform into hydrogels. Finally, the crosslinked hydrogels were immersed in water for three days to remove any remaining residue. A series of GO/cellulose hydrogels were obtained with various GO weight contents (GO:cellulose = *x*:100, where *x* = 2.5, 5, 10, 20, and 30). The resultant hydrogels were labeled as GO(*x*)/cellulose(100).

### 3.4. Characterization

Fourier transform infrared (FTIR) spectra of the dried hydrogels were recorded with a Thermo Scientific Nicolet iN 10 FTIR Microscopy instrument (Thermo Nicolet Corp., Madison, WI, USA) equipped with a liquid nitrogen-cooled mercury-cadmium-teluride (MCT) detector. The scan range was 600–4000 cm^−1^, and the distinguishability was 2 cm^−1^. X-ray diffractograms were collected on an XRD-6000 instrument (Shimadzu, Kyoto, Japan) with an incident wavelength of 1.54 Å (Cu Kα radiation) and a detector at a scanning rate of 1 min^−1^ over the 2θ range, from 5° to 45°.

Cellulose hydrogels were weighed (*M*_h_) and then dried at 105 °C to a constant weight. The dried sample was cooled down in a desiccator to room temperature and weighed (*M*_d_). The water content (*W*_c_) can be calculated as:

*W*_c_ = 100 − *M*_d_/*M*_h_ × 100 (5)

The compressive test was performed on cellulose hydrogels at a rate of 5 mm·min^−1^ by a CMT6503 Test Machine (ShenZhen SANS, Shenzhen, China). The undried hydrogel samples were cylindrical hydrogel 5.0 mm in diameter and 5.0 mm in thickness.

The Brunauer-Emmett-Teller (BET) was measured with a Tristar II 3020 instrument (Micrometrics Instrument, Norcross, GA, USA), using the adsorption of N_2_ at the temperature of liquid nitrogen. Prior to measuring, all of the samples were degassed at 393 K for 16 h and finally outgassed to 10^−3^ Torr. All of the samples were tested three times and the the average value was used.

The morphologies of hydrogels were examined using scanning electron microscope (SEM) instrument (Hitachi S-3400N II, Tokio, Japan). All hydrogel samples were immersed in distilled water at room temperature and allowed to swell to equilibrium, then fast-frozen in liquid nitrogen, and freeze-dried before SEM observation.

The method for determination of the point of zero charge (pH_pzc_) was proposed by Balistrieri and Murray. Accordingly, to a series of well-stoppered 100 mL polyethylene bottles containing 40 mL of aqueous sodium nitrate solutions, different amounts of either 0.1 M HCl or 0.1 M NaOH solution were added in order for the pH of the samples. The bottles were filled to 50 mL with the aqueous sodium nitrate solutions. After 2 h of equilibration the pH values were noted as pH*_i_*. A known amount of hydrogel was added in each bottle and left at 30 °C for 72 h with shaking. The pH values of the supernatant liquid in each bottle was noted as pH_f_. 

### 3.5. Adsorption Studies

#### 3.5.1. Preparation of Cu^2+^ Solution

Cu^2+^ solutions (500 mg·L^−1^) were prepared by dissolving 1.9644 g solid CuSO_4_·5H_2_O in 1000 mL of deionized (DI) water. The other solutions of different concentrations were adjusted by serial dilution.

#### 3.5.2. Adsorption Procedures

Unless otherwise stated, batch experiments were carried out (at 298 K) by agitating a fixed mass of dry hydrogel (10 mg, the GO(20)/cellulose(100)) in 50 mL of metal solutions (initial Cu concentration of 200 mg/L, initial pH of solution 5.3) at 100 rpm for 120 min. The adsorbent/heavy metal ion solution mixtures were shaken in a thermostatic oscillator (Labwit Scientific, Shanghai, China). The supernatant was transferred for determination of Cu^2+^ concentration by measuring the absorbance at 810 nm (Abs_810_) [37] using a UV 2300 spectrophotometer (Techcomp, Shanghai, China). Preliminary experiments showed a linear correlation between Abs_810_ and Cu^2+^ concentration. All of the samples were tested three times and the the average adsorption intensity was used to estimate Cu^2+^ concentrations. The equilibrium absorption amount of metal ions absorbed on the bioadsorbent, *q*_e_ (mg/g), was calculated using Equation (6):

*q*_e_ = (*C*_0_ − *C*_e_)*V*/*m* (6)

where *C*_0_ is the initial metal ions concentration (mg/L), *C*_e_ is the equilibrium metal ions concentration in solution (mg/L), *m* is the weight of the dried hydrogel used (g), and *V* is the volume of the metal ions solution (L).

Kinetics experiments were carried out with different initial Cu(II) concentrations (50 and 100 mg/L), and the mixture was agitated continuously for 3–150 min. To study the effect of temperature, isothermal experiments were conducted at 293, 298, and 303 K. In this group of experiments, the initial Cu(II) concentration was varied from 50 to 400 mg/L.

#### 3.5.3. Desorption and Reusability Behaviors of GO/Cellulose Hydrogel

After the attainment of equilibrium, the Cu^2+^-loaded hydrogel was filtered from the solution and washed several times with distilled water to remove any unabsorbed Cu^2+^. Thereafter, the bioadsorbents were immersed into 0.1 M HCl solution (50 mL) for 2 h to remove the adsorbed Cu^2+^ from the hydrogel and then regenerated with 0.1 M NaOH for 1 h. Finally, the hydrogel particles were thoroughly washed with deionized water to reach a neutral pH and again used in the adsorption experiment. The desorption efficiency was calculated according to Equation (7):

(7) $$\text{desorption efficiency}=\frac{\text{amount of Cu}\left(\text{II}\right)\text{ desorbed}}{\text{amount of Cu}\left(\text{II}\right)\text{ absorbed}}\times \;100\%$$

#### 3.5.4. Adsorption of Other Hazardous Metals

Ten milligrams of dried hydrogel were soaked in 50 mL of 100 mg/L multi-metal (Zn + Fe + Pb) solutions. The mixtures were shaken in a thermostatic oscillator at 100 rpm for 120 min at 298 K. The heavy metal ion concentration of the supernatant liquid was determined using an inductively coupled plasma optical emission spectroscopy (Optima 8x00, PerkinElmer, Foster City, CA, USA) for Zn^2+^, Fe^3+^, and Pb^2+^.

## 4. Conclusions

A novel and easy method has been proposed to prepare cellulose/GO hydrogel with good adsorption of heavy metal ions from aqueous solutions. FTIR and XRD measurements indicated the existence of crosslinking reaction between the GO and the cellulose matrix. The incorporation of GO increased the compressive strength of the GO/cellulose hydrogel and significantly improved their adsorption capacities for the metal ions. The adsorption capacity of Cu^2+^ increases with an increase in the GO/cellulose ratio, while the adsorption capacities decreased continuously with an increasing dosage of GO/cellulose hydrogel. The adsorption kinetics data could be well described by the pseudo-second-order model, and the adsorption process followed the Freundlich isotherm model. In addition, GO/cellulose hydrogel exhibited excellent reusability and also substantially adsorbed other harmful metal ions (Zn^2+^, Fe^3+^, and Pb^2+^). This study provided a highly efficient bioadsorbent for the removal of heavy metals from an aqueous solution.

## Figures and Tables

**Table 1:** Water content, compressive modulus, surface area, pore volume, and pore size of GO(*x*)/cellulose(100) hydrogels with *x* = 2.5, 5, 10, 20, and 30, respectively.

Sample	2.5	5	10	20	30
Water content (wt %)	92.7	90.3	94.1	92.5	93.5
Compressive modulus (kPa)	114	193	144	128	115
*S*_BET_ (m^2^·g^−1^)	0.19	13.41	40.72	25.11	45.12
Pore volume (cm^3^·g^−1^)	0.0023	0.0575	0.1807	0.0048	0.1856
Pore size (nm)	3.11	7.49	7.08	5.15	6.73

**Table 2:** Comparison between the pseudo-first-order and pseudo-second order kinetic models for Cu^2+^ sorption onto GO(20)/cellulose(100) hydrogel.

*C*_0_ (mg·L^−1^)	Pseudo-First-Order Model			Pseudo-Second-Order Model		
	$q_{e}^{\mathrm{cal}}$ (mg·g^−1^)	*k*_1_ (g·mg^−1^·min^−1^)	*R*^2^	$q_{e}^{\mathrm{cal}}$ (mg·g^−1^)	*k*_2_ (g·mg^−1^·min^−1^)	*R*^2^
50	16.44	0.0106	0.876	37.59	0.0023	0.991
200	46.92	0.0187	0.846	94.34	0.0009	0.998

**Table 3:** The parameters for Langmuir and Freundlich models for Cu^2+^ sorption onto GO(20)/cellulose(100) hydrogel.

T (K)	Langmuir			Freundlich		
	*Q*_max_ (mg·g^−1^)	*b* (L·mg^−1^)	*R*^2^	*k* (mg^1−1/n^·L^1/n^·g^−1^)	*n*	*R*^2^
293	138.888	0.014	0.9552	18.01	3.19	0.9703
298	384.615	0.001	0.9406	0.92	1.19	0.9982
303	192.308	0.004	0.8098	1.72	3.78	0.9633
